# How methodological changes have influenced our understanding of population structure in threatened species: insights from tiger populations across India

**DOI:** 10.1098/rstb.2020.0418

**Published:** 2022-06-06

**Authors:** Megan Aylward, Vinay Sagar, Meghana Natesh, Uma Ramakrishnan

**Affiliations:** ^1^ National Centre for Biological Sciences, TIFR, Bangalore, India, 560065; ^2^ Indian Institute of Science Education and Research, Tirupati, India, 517507; ^3^ Senior Fellow, DBT Wellcome Trust India Alliance, Hyderabad, Telangana, India, 500034

**Keywords:** genomes, SNPs, genetic markers, tiger, conservation, endangered species

## Abstract

Unprecedented advances in sequencing technology in the past decade allow a better understanding of genetic variation and its partitioning in natural populations. Such inference is critical to conservation: to understand species biology and identify isolated populations. We review empirical population genetics studies of Endangered Bengal tigers within India, where 60–70% of wild tigers live. We assess how changes in marker type and sampling strategy have impacted inferences by reviewing past studies, and presenting three novel analyses including a single-nucleotide polymorphism (SNP) panel, genome-wide SNP markers, and a whole-mitochondrial genome network. At a broad spatial scale, less than 100 SNPs revealed the same patterns of population clustering as whole genomes (with the exception of one additional population sampled only in the SNP panel). Mitochondrial DNA indicates a strong structure between the northeast and other regions. Two studies with more populations sampled revealed further substructure within Central India. Overall, the comparison of studies with varied marker types and sample sets allows more rigorous inference of population structure. Yet sampling of some populations is limited across all studies, and these should be the focus of future sampling efforts. We discuss challenges in our understanding of population structure, and how to further address relevant questions in conservation genetics.

This article is part of the theme issue ‘Celebrating 50 years since Lewontin's apportionment of human diversity’.

## 1. Introduction

Population genetics investigates variation in the wild and allows quantification of micro-evolutionary change in allele frequencies over time. Starting out as a theoretical discipline, by the 1950s, population genetics was rich in theory, with little empirical data. Richard Lewontin was among the early population geneticists who worked to bridge this gap [1,2]. Lewontin and his colleagues used allozymes to quantify genetic variation and aimed to understand the distribution of genetic variation in humans. By the late 1990s, the invention and routine use of Sanger sequencing and PCR (in the 1970s and 1980s, respectively) allowed the creation of molecular genetic datasets to test population genetic patterns using Sanger sequence data of short DNA regions and later, highly polymorphic microsatellite markers. More recently, the advent of high-throughput sequencing (HTS) allowed the generation of large datasets at relatively reasonable costs [3]. These technological advances, as well as the associated analytical methods, have shaped the growth of empirical population genetics, and expanded its scope from humans and model organisms to the investigation and study of non-model organisms, wild populations (e.g. [4]) and endangered species (e.g. [5]).

In parallel with advances in sequencing technology, our planet's biodiversity is experiencing pervasive population and species decline (e.g. [6,7]). Such decline has been accompanied by the burgeoning of conservation biology [8,9], the discipline that deals with biological correlates and drivers of extinction. Decades of theoretical, experimental and empirical research suggests that small and isolated populations are more likely to go extinct because of demographic, environmental and genetic stochasticity. Genetic stochasticity, or drift, leads to the loss of genetic variation in small and isolated populations (e.g. [10]), and such loss, especially when involving adaptive alleles, could accelerate extinction. The field of conservation genetics has been shaped by Lewontin's ideas in understanding how genetic diversity is distributed within a species. Understanding population structure is particularly important for endangered species, where conservation management strategies depend on accurate detection of species status, habitat connectivity, inbreeding, adaptive variation and population demographic history [11]. Studies of population structure are often used to identify isolated populations (e.g. [12]). Through the study of population structure, we can begin to ascertain historic connectivity, which can allow inference of habitat loss and local extinction of past populations. This information is fundamental in assessing the risk of extinction and in attempts to restore population connectivity that has been lost. The field of conservation genetics started with studies mainly describing population genetic structure for endangered species (e.g. the red-cockaded woodpeckers [13]). The scope of population genetics investigations of endangered species was largely defined by a series of papers published in the early 1990s [14–18].

Detection of population structure is potentially challenging, especially if such structure is recent, or associated with changes in population size (e.g. [19]). For many endangered species, the loss of habitat and population size decline have occurred relatively recently, in the past few hundred years (e.g. [20]). Detection of such shallow population structure tends to require large genetic datasets and/or broad spatial sampling (e.g. [21]). Technological breakthroughs led to the possibility of higher statistical power through the use of several thousands to millions of markers, and the burgeoning of conservation genomics [22] and landscape genetics (e.g. see [23]). Studies of population structure are often used to identify isolated populations (e.g. [12]). More recently, attempts are being made to incorporate genetic variation, its partitioning and its loss into legal and international agreements, such as the Convention on Biological Diversity (e.g. [24]).

While understanding of population structure, connected landscapes and the history of isolated populations continues to be significant, questions regarding their conservation, the spatial scale, sampling strategy and genetic data involved have changed over time. Study design—including sample size, where to sample, and how many markers to use—depends on the questions that are of conservation concern. Additionally, in conservation biology, other considerations influence how studies are designed, such as capacity to conduct genomic studies based on infrastructure available to process samples, generate data and analyse large datasets, and available funding [25]. Several studies have used empirical data and simulations to investigate impacts of study design (e.g. [26]), marker type (e.g. [27,28]) and time since population differentiation [29] on inferences of population structure. Overall, population genetic theory suggests that our ability to understand population structure is best when we have high sample size and many markers because these increase the number of alleles that are sampled and thus increase the power of analysis, but when resources can only be allocated to one type of sampling effort, increasing the number of markers might be better than only increasing sample size [30]. In population genetics power is often a factor of the number of alleles sampled. Although this does not scale linearly, a focus on increasing numbers of markers and moving towards genomic-scale approaches will increase number of alleles by orders of magnitude, which cannot be achieved through only sampling more individuals.

**Figure 1.**
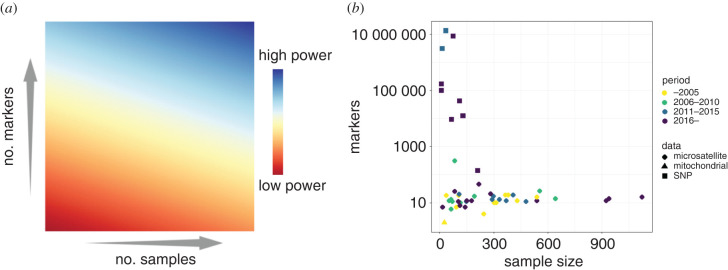
(*a*) Schematic showing the power to infer population structure as a function of sample size and the number of markers. (*b*) Actual numbers of markers (represented categorically) and sample size for a set of studies investigating population structure in species of large mammals (see electronic supplementary material, table for studies and species).

Figure 1*a* presents a schematic of a possible broad-brush understanding of how markers and sample size interact to provide power to inference of population structure. Figure 1*b* shows sample size and number of markers used in studies investigating population structure for large-bodied mammals with slow life histories (primarily endangered primates and carnivores). The studies selected for the plot include a non-exhaustive list of species with population genetics data spanning the last 20 years to highlight any changes in sampling approaches over time in endangered species. This includes both range-wide and regional studies. Details of the studies are in electronic supplementary material, table S1.

Overall, we are seeing an increase in conservation genetic studies from the lower power quadrant in figure 1*a* of few samples and few markers to the higher power quadrant, with an increase in the number of markers for conservation genomics approaches. However, this shift from genetics to genomics scale approaches is not present in all cases; many studies continue to use fewer than 100 makers, although of these some benefit from high sample size. While methods for obtaining genome-wide data from non-invasive samples are improving (see [31–33], such samples are affected by DNA degradation and low amounts of DNA from the target species—which makes achieving sufficient coverage and high-quality genotype calls across the whole genome less feasible. Genomic studies emerged relatively early for ape species compared to carnivores, likely reflecting the availability of reference genomes (driven by interest in human evolution), which continues to be a consideration in population genomic studies of endangered species.

Conservation genetic studies of tigers (*Panthera tigris*) have been ongoing since 2004 [34]. Tigers represent the largest terrestrial carnivore on earth and are a flagship species for conservation globally. Tigers range across much of south and southeast Asia and include six extant subspecies. Despite their broad distribution, tigers are threatened with extinction across their range. Habitat loss, fragmentation and hunting threaten all tiger subspecies. Although tigers range across fourteen countries, around two-thirds of extant tigers live in India [35]. Range-wide genomic data suggest tigers in India harbour substantial genetic variation and are a distinct population. They belong to the previously described subspecies *Panthera tigris tigris*, known as Bengal tigers [36]. Together, these factors make it important to understand population structure for tigers in India and make them an ideal species for assessing how changes in methods have influenced population genetic studies in endangered species. Tigers are large carnivores, and we expect them to disperse long distances. Long-range dispersal, on the order of 384 km, has been observed for individual tigers (e.g. [34]) and has been suggested by landscape genetic studies [35]. Given that tigers can disperse long distances, we do not expect a strong population structure, at least at short spatial scales. Further, range-wide demographic history estimates suggested relatively recent divergence (in the past few thousand years), even between tiger subspecies. Finally, landscape genetic studies suggest that high traffic roads and high-density human settlements are the only factors detrimental to tiger connectivity [37]. Taken together, it appears likely that we may not see strong signatures of population structure within the Indian subcontinent, especially at small spatial scales. However, a combination of short generation time and small population sizes for tigers means it may be possible to detect the effects of these anthropogenic features at a fine spatial scale in some populations.

In the pioneering study on tiger phylogeography in 2004, Luo *et al*. [34] used mitochondrial haplotype data (4 kb), nuclear microsatellites [30] and the MHC class II DRB region to understand population genetics of all tiger subspecies. This study established the basis for tiger population genetics, showing that different populations and subspecies are genetically distinct. Since this first study of all tiger subspecies across their range, research and the approaches used have advanced, along with developments in genetic techniques such as marker development and sampling methods. Population genetics studies of tigers have used an array of available markers to understand population structure at multiple spatial scales, from regional studies to the whole range, and from mtDNA to whole genomes.

Here we use Bengal tigers as a case study to review how our understanding of population structure has changed with different marker types, sequencing technologies and sampling strategies over time. We assess how these developments can benefit the application of population genetics to conservation of wild species. We include our own analyses that use data from complete mitochondrial genomes (mitogenomes), a single-nucleotide polymorphism (SNP) panel and whole genomes, and we explore how our new analyses fit with previously published studies. We show how the field has expanded from mitochondrial markers, to microsatellites, to genome-wide SNP variants, and how this change has informed inference over time. Finally, we discuss gaps that exist in our understanding of population genomics and structure for Bengal tigers and the importance of this information in conservation management.

## 2. Methods

In our review, we included studies that have sampled Bengal tigers across India to assess population structure (table 1). The studies published to date include two studies that have used microsatellite data [40,41] and one that used 10 184 genome-wide SNPs generated using restriction site-associated DNA markers (RAD-seq) [12]. To gain a fuller understanding of how inferences change depending on scale of sampling and marker types used, we conducted three new analyses using (i) mitochondrial haplotypes, (ii) 194 824 genome-wide SNPs and (iii) a panel of 81 SNPs. These additional analyses allow us to further compare nuclear versus mitochondrial markers and SNP versus microsatellite markers, while also assessing the impact on the number of markers and number of populations sampled. Whole-genome data (including both nuclear and mitochondrial sequence data) were available from three published studies; two that assess population structure across all tiger subspecies [36,39] and one on assessing inbreeding among tiger populations within India [38]. We used SNP panel data from [10], which used an extensive dataset of primarily non-invasive samples to screen for the taqpep locus mutation for pseudo-melanism within Bengal tigers as part of a panel of 123 SNP loci.

**Table 1.** The empirical datasets reviewed in this study on population structure in Bengal tigers. We provide information on the marker types, sampling schemes and data sources used in the presented analyses. For nuclear DNA the number of loci corresponds to actual number of variants used, whereas for mitochondrial DNA number of loci is the total number of base pairs sequenced.

DNA type	type of variants	number of loci	number of individuals	number populations sampled	data source	analysis
nuclear	SNP	198 930	37	8	Khan *et al*. [38]	this study
					Armstrong *et al*. [36]	
					Liu *et al*. [39]	
nuclear	SNP	10 184	38	15	Natesh *et al*. [12]	Natesh *et al*. [12]
nuclear	SNP	81	155	24	Sagar *et al*. [10]	this study
					Khan *et al*. [38]	
					Armstrong *et al*. [36]	
					Liu *et al.* [39]	
nuclear	microsatellite	11	158	34	Kolipakam *et al.* [40]	Kolipakam *et al.* [40]
nuclear	microsatellite	8	56	28	Mondol *et al*. [41]	Mondol *et al*. [41]
mitochondrial	sequence	15435 bp	42	4	Khan *et al*. [38]	this study
					Armstrong *et al*. [36]	
					Liu *et al*. [39]	
Mt	sequence	1200 bp	57	27	Mondol *et al*. [42]	Mondol *et al*. [42]
Mt	sequence	932 bp	77	13	Sharma *et al*. [43]	Sharma *et al*. [43]

### (a) Whole genomes

#### (i) Data processing and variant calling

**Figure 2.**
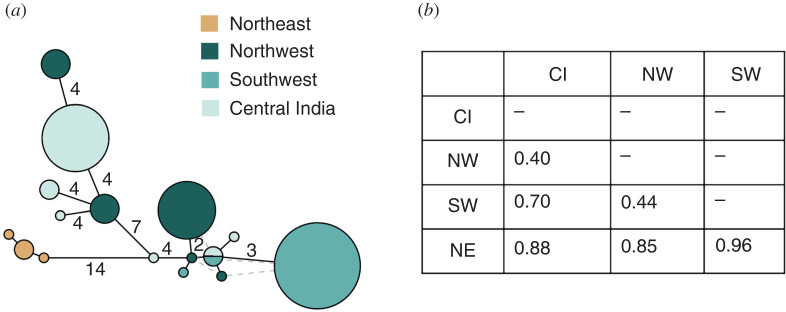
Mitogenome analysis using 42 individuals (dataset table 1, row 6) from four regional populations: CI (*n* = 13), NW (*n* = 14), SW (*n* = 11) and NE (*n* = 4). (*a*) Maximum-parsimony network for four regional populations of tigers using mitochondrial genomes minus the control region. Numbers on the network indicate the number of mutations between the nodes; lines without a number indicate only one mutation. Circle size is proportional to the number of individuals with that haplotype. (*b*) Pairwise *F*_st_ among all four populations CI, NW, SW and NE, calculated from mitogenomes using PopGenome in R, v. 3.4.2 [55,56].

For analysis of whole-genome and mitochondrial genome population structure, we used data from six populations and 59 individuals across India generated by [36,38,39,44] available on NCBI under accessions PRJNA559670, PRJNA728665, PRJNA693788 and PRJNA437782, respectively. These data represent broad-scale sampling from multiple regions across India with the exception of the southeast. These areas include northwest (NW) (*n* = 20), southwest (SW) (*n* = 15), Central India (CI) (*n* = 13), north (*n* = 3), northeast (NE) (*n* = 5) and Sundarbans (SU) (*n* = 3) (figure 2*a*). We filtered and trimmed fastq data for each sample using prinseq lite [46] with the following criteria; minimum length 120 bp, minimum mean quality 30. We repaired fastq files using bbmap [47] repair.sh and kept only paired reads. We aligned paired reads to the reference genome assembly for *P. tigris tigris* (available on NCBI under accession GCA_021130815.1) using bwa-mem [48]. We removed duplicate reads using Picard tools Mark Duplicates command [49].

We called variants in the same way for both mitogenomes and nuclear markers but set the ploidy to one for the mitogenome. We called variants using GATK-4.1.7.0 using haplotypeCaller, CombineGVCFs and GenotypeGVCFs commands [50,51]. We filtered variant sites to retain those with a minimum quality of 30 and filtered genotypes to keep those with minimum depth of 10 and minimum genotype quality of 30, and retained only biallelic SNPs using GATK-4.1.7.0 SelectVariants and FilterVariants commands. For the mitogenome analysis, we selected only the reads aligning to the mitochondrial genome using Samtools-view command [52] and called variants separately from the nuclear DNA. By aligning to the whole genome rather than only the mitochondrial genome, we reduced the potential occurrence of numts as these reads should preferentially align to the nuclear regions.

#### (ii) Mitogenome network

We further filtered the mitogenome dataset to remove any individuals with less than 50% coverage and removed sites where fewer than 90% of individuals were genotyped. We excluded any population with fewer than four individuals sampled. This filtering resulted in a final mitogenome dataset of 42 individuals. We included complete mitogenomes (without the control region as this will show finer-scale differences rather than revealing regional-scale differences) from four regions—NW, SW, CI and NE India—in our analysis. We conducted analysis of haplotypes from the variant call format (VCF) file using VCFR [53] and Pegas [54] packages in R v. 3.4.1 [55]. We generated a maximum-parsimony network to assess the number of haplotypes and the distribution of these among sampling locations. We calculated *F*_st_ among the four regions using the PopGenome package [56] in R, v. 3.4.1 [55].

#### (iii) Whole-genome population structure

We selected the 18 nuclear chromosome regions (excluding the mitogenome, sex-chromosomes and unplaced scaffolds) from the filtered whole-genome VCF file. We further filtered to remove any site with more than 80% missing data, then removed any individuals with more than 50% missing data after initial filtering to eliminate individuals that had low coverage across the genome. This resulted in 46 individuals. Finally, we removed all loci with more than 10% missing data; all loci were represented by at least 90% of individuals to ensure there were no loci that might have missing data for a particular region as this could bias our analysis of population structure. We filtered sites for Hardy-Weinberg equilibrium value of 0.0001 using VCFtools v. 1.13 [57]. We removed sites that were in high linkage disequilibrium (LD) using PLINK v.1.9 [58] with an *r* value > 0.8, a window size of 50 and step size of 10. Given the potential for highly related individuals within these populations of tigers, we calculated relatedness between pairs of individuals. We calculated relatedness using the ‘genome’ function in PLINK v.1.9 and removed one individual from any pair with relatedness greater than 0.5, which resulted in the removal of nine individuals. After filtering, 198 824 SNPs and 37 individuals were retained and used for the analysis of population structure. We used ADMIXTURE [59] to estimate individual ancestry for values of *K* from 2 to 7, using the default parameters. ADMIXTURE uses a similar algorithm to STRUCTURE but is less computationally intensive and runs efficiently on large datasets. Although STRUCTURE uses a Bayesian approach and ADMIXTURE implements a maximum-likelihood approach, these programs explore the same parameter space and use the same assumptions. However, ADMIXTURE does not account for LD between loci. Our filtering to remove loci that were in high LD in the whole-genome datasets addresses this issue to make our results comparable with analyses that have used the STRUCTURE program [59,60]. We used the covariance measure to predict optimal *K*. We plot the ADMIXTURE output using the pophelper [61] package in R, v. 3.4.1 [55].

### (b) Single-nucleotide polymorphism panel

#### (i) Data processing and genotyping

For the SNP panel analysis, we used data generated by Sagar *et al*. [10] for a panel of 123 nuclear DNA SNP loci that are known to be polymorphic in Bengal tigers across India [62], for 133 individuals primarily from non-invasive samples. These data were Fastq files obtained from multiplex PCR amplification (mPCR) of target loci followed by HTS. We used TrimGalore (see https://github.com/FelixKrueger/TrimGalore) to trim the raw fastq files to remove adaptor sequences and low-quality reads with a minimum quality of 30 and a stringency value of 5. We aligned the retained reads to the reference Bengal Tiger Genome (JAHFZI000000000, NCBI) using bwa-mem [48] (mismatch penalty 3) and called variants using bcftools v. 1.11 [52]. We used GATK v. 4.1.0 to mark any genotype with a quality of less than 10 (GQ < 10) and a depth of less than 10 (DP < 10) as missing. In addition to samples genotyped using mPCR, we included all 59 individuals from the whole-genome nuclear data cited above. The raw whole-genome fastq files were processed to produce a VCF file as described in [40]. We extracted the 123 nuclear SNP loci of the SNP panel from this VCF of genome-wide variants. We combined the two VCF files, one from mPCR sequence data and one for whole genomes into one VCF file using bcftools v1.11 -merge command. We filtered the VCF file using VCFtools for minimum genotype quality (GQ 30), minimum quality (Q 30) and minimum depth (DP 10). We removed any individual with more than 50% missing data. We filtered SNPs to remove any loci with fewer than 60% of individuals genotyped at that locus, which left 81 SNPs. We calculated the relatedness between all pairs of individuals in the merged VCF using the ‘genome’ function in PLINK v.1.9. We observed that five pairs of individuals had a relatedness value of more than 0.8 (Pihat > 0.8). We removed one individual from each such pair to avoid including the same individual or highly related individuals twice. Our final SNP panel dataset included 175 individuals; 46 from the whole-genome dataset and 129 from mPCR data.

#### (ii) Single-nucleotide polymorphism panel population structure

We converted the VCF file to STRUCTURE v. 2.3.4 format using PGDSpider v. 2.1.1.5 [63]. We ran one million Markov chain Monte Carlo repeats with a burn-in period of 50 000 for *K* = 2–10, with 10 repeats for each value of *K*. The range of *K* was chosen based on the maximum likely number of clusters (plus one) given previous studies of population structure in tigers, barriers to gene flow and recorded dispersal distance of tigers. The results of STRUCTURE were analysed and plotted using the pophelper package in R, v. 3.4.1 [55,61].

## 3. Results and discussion

### (a) Mitochondrial DNA

The earliest population genetics study of tigers by Luo *et al*. [34] revealed low nucleotide diversity within the Bengal tiger subspecies relative to all other tiger subspecies, whereas Mondol *et al*. [41] reported the highest diversity in Bengal tigers relative to these other subspecies. More recent analysis of whole mitogenomes by Liu *et al*. [39] supported Mondol *et al*. [41] and indicated that Bengal tigers harbour the highest nucleotide diversity of all tiger subspecies.

In addition to the effect of sample size and spatial scale of sampling on inferences of population structure, how researchers chose to group populations in analyses also affects inferences. Two separate studies of tiger mitochondrial DNA in south Asia by [42,43] indicated that populations in the north (Nepal and the Terai landscape) are distinct to those in SW and in CI. Mondol *et al.* [42] and Sharma *et al*. [43] used 57 samples of 1200 bp and 77 samples of 932 bp mitochondrial genome, respectively. However, despite similar scales of sampling and including some of the same mtDNA genes, the *F*_st_ values among populations in these studies vary immensely. Mondol *et al*. [42] report maximum *F*_st_ of 0.298 (between north and south India), whereas Sharma *et al*. [43] report much greater structure, with *F*_st_ values of up to 0.88 between north and other regions (but specific pairwise values are not reported). These two studies use a different grouping of populations to represent regions; Mondol *et al*. [42] use much coarser regional grouping than Sharma *et al*. [43]. They group populations into three regions (north, central and south), whereas Sharma *et al*. [43] separate populations in the NW from those in north India and further divide the northern region into three populations, with east to west divisions (northern India in the west, southern Nepal in the centre and a NE population–east of Bangladesh). Therefore, the *F*_st_ values may be reduced or increased based on this clustering, whereby grouping distinct populations into one cluster may dilute the signal of population structure when compared to a different cluster.

We include our own analysis of complete mitochondrial genomes. We included four broad geographical regions, CI, NW, SW and NE, which have at least four individuals sampled per population. This grouping is most similar to that of Sharma *et al*. but does not include populations representing northern India and southern Nepal.

Our mitogenome analysis indicated that the NE population is most distinct, with high *F*_st_ (0.85–0.96) compared to pairwise comparisons among each of the three other regions. The SW population also has relatively high structure, with CI (*F*_st_ 0.7), whereas CI and NW have more dispersed haplotypes in the network and the lowest pairwise *F*_st_ (0.40). The pattern here supports what has been shown in demographic history analysis, whereby the NE population is predicted to have diverged prior to all other populations [36]. Although the NE population has a different demographic history from CI, whether there continues to be gene flow between the NE and CI is not clearly resolved. The relatively dispersed haplotypes in the NW population is surprising given that there are high rates of inbreeding within this one population [38], and it has experienced multiple, severe bottlenecks in the recent past [64]. However, this could indicate a few founder lineages that have persisted. Additionally, the divergence between the NW population and CI populations is likely very recent and therefore despite acute population bottlenecks in the past 50 years in the NW population, some of this diversity may remain [65]. This sharing of haplotypes between CI and NW India is also seen in Sharma *et al*. [43], whereas Mondol *et al*. [42] group the NW individuals with populations that are classified as the north in Sharma *et al*. [43]. This clustering could explain the much lower *F*_st_ values among populations in Mondol *et al*. [42] relative to both our study and Sharma *et al*. [43]. The degree of structure that we observe between SW and CI is high and does not reflect the pattern of shared haplotypes that Sharma *et al*. [43] showed. However, using whole-mitochondrial genomes rather than a few genes allowed the inclusion of more variants and therefore the potential to detect diversity among geographic regions. Conversely, the high *F*_st_ values in our study overall could be a result of the limited sampling at the population level; each region is only represented by one population. Given the non-recombining nature and matrilineal mode of inheritance, it is possible that much of the within-region diversity is not captured with a limited sampling strategy. Despite the relative ease in amplifying and obtaining mitochondrial DNA, the extent of sampling of mitochondrial markers is relatively limited. To truly understand mitochondrial population structure, it is important to extend sampling and combine existing datasets, and then to assess population structure without prior assumptions or population clustering.

### (b) Nuclear markers

The analysis of population structure can be confounded by the study design, uneven sampling, degree of population differentiation and population histories [66,67]. Mondol *et al.* [41] were the first to use nuclear DNA to investigate population structure within Bengal tigers across India. They used eight microsatellite markers genotyped in both historic and present-day samples to assess change in connectivity and diversity over time and revealed a reduction in connectivity over the past 200 years. To understand the structure of present-day populations across their range in India, recent studies have used a variety of sampling strategies. These studies differ in terms of markers used (microsatellites or SNPs), the number of loci genotyped and scale of sampling—both in terms of sample size and spatial representation. Three recent studies have analysed the India-wide genetic structure of Bengal tigers: (i) Natesh *et al*. [12] used invasive samples of tigers (*n* = 54) collected opportunistically to generate RAD-seq data on SNP loci (10 184)—low sample size, many markers; (ii) Kolipakam *et al*. [40] used non-invasive samples of tigers (*n* = 158) to generate genotype data on 11 microsatellite markers—higher sample size, fewer markers; and (iii) Armstrong *et al*. [36] used invasive samples of Bengal tigers (*n* = 21) as part of a larger dataset of all extant subspecies, to generate genome-wide sequence data—low sample size, large number of markers. We present two additional investigations of pan-India population structure in Bengal tigers. Our analyses include (i) whole-genome sequences from 37 individuals and (ii) 81 SNPs genotyped from 175 individuals—129 non-invasively sampled individuals and 46 individuals from the whole-genome dataset with coverage at these loci. We compare these studies to highlight agreement and disparities based on the sampling strategy and how these shape our understanding of tiger population structure.

SNP-based studies, whether based on whole genomes or fewer than one hundred SNPs, infer three predominant population clusters across India at *K* = 3 (figure 2). This suggests that the SW, NW and those in central-east–north-northeast populations are differentiated. This inference is important for conservation management of tigers as it indicates that these populations have potentially experienced different evolutionary trajectories in the recent past. However, the SNP panel analysis with 81 loci shows that two populations in the far south of the SW cluster with CI. These populations were not sampled in any of the other SNP-based studies. Generally, this area is relatively under-sampled across studies, likely as a result of the very low density of tigers in this area and therefore challenges in sampling [35]. It is possible that genetic drift may have fixed variants in these small southern populations and the affinity to CI is because this region has the highest genetic variation [38]. Alternatively, because only four individuals were sampled from these populations, low sample size and relatively low numbers of markers may have resulted in low power to accurately determine the clustering of these two populations.

**Figure 3.**
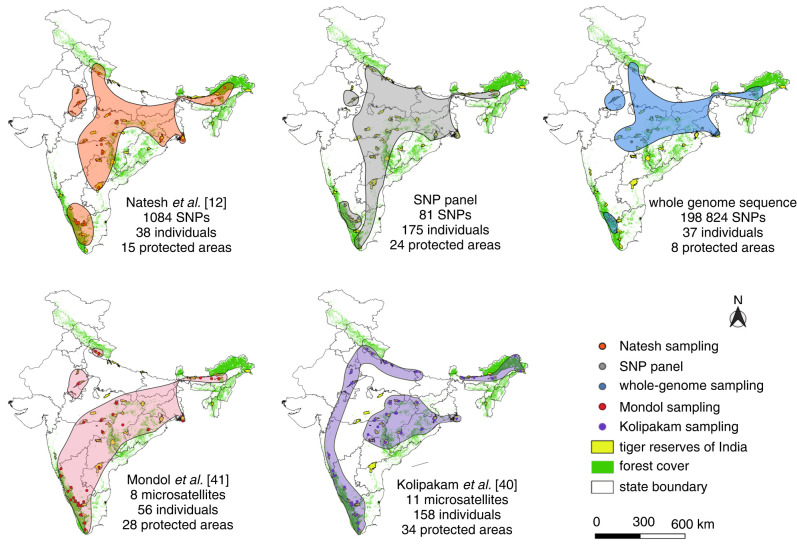
An overview of the nuclear DNA studies in Bengal tigers to date reveals population structure as inferred based on population structure analysis of these populations at optimal *K*. We include three previously published studies (table 1, rows 2, 4, and 5) [12,38,39] and the two nuclear marker analyses in this paper. We show this in the context of assumed differences in power to detect structure based on the sampling scheme (number of markers and number of individuals) and number of areas sampled as the number of protected areas.

In figure 3, we summarize the broad inferences of tiger population structure as assessed through five datasets, including the ones presented here, at the preferred values of *K* based on the Evanno method [45]. The consistent pattern of regional clustering that we see among SNP-based studies was not reflected in microsatellite studies. Kolipakam *et al*.'s [40] study based on microsatellite markers showed two key disparities with the SNP-based studies: (i) while all SNP-based analyses revealed that the NE population clustered with CI until at least *K* = 4, Kolipakam *et al*. [40] reported that the NE is the first genetic cluster to separate from the other populations; (ii) Kolipakam *et al*. [40] reported that the NW population did not differentiate from north India even at high values of *K* (up to 10), while the three SNP-based studies and Mondol *et al*. [41] show that the NW population was the first to form a separate cluster at *K* = 2, and the north population clustered with CI up to *K* = 4. Despite the inferred grouping in Kolipakam *et al*. [40], the structure plots do not show distinct clusters. Even at *K* = 3, some CI individuals, particularly those from Kanha/Pench and Orisa, show high proportions of ancestry shared with the NE. As Kanha tiger reserve is the population that was sampled for the whole-genome analysis, the clustering that we see could be due to the greater amount of shared ancestry between Kanha and the NE compared to other CI populations. However, Natesh *et al*. [12] sampled multiple populations within CI at over 10 000 loci and showed that these populations cluster together and do not show different ancestries (figure 3). Mondol *et al*. [41] did not reveal differentiation between the SW and CI, rather reported three clusters of north, NW and central-south–northeast. The disagreement between the two microsatellite studies [40,41] may be due to the lower sample size in Mondol *et al*. [41] (*n* = 57) compared to Kolipakam *et al*. [40] (*n* = 158), yet the spatial distributions of samples are similar. Alternatively, the difference may be due to different markers used in the two different studies (only two microsatellite loci overlapped), which highlights the impacts of marker selection/availability on inferences when using microsatellite markers.

SNP-based markers may reveal more distinct clusters with lower amounts of admixture relative to microsatellites (e.g. [68–71]). This may be due to the increased power and lower error in genotyping SNP markers [68,72]; typically, only biallelic SNPs are retained for analysis from HTS data, and the variants can be filtered to remove any low-quality genotypes or loci. Of the studies reviewed in this paper we see this pattern; population clustering is more distinct in the SNP-based studies relative to microsatellite studies. In Kolipakam *et al*.'s [40] microsatellite study, at the country-wide scale, only the NE samples form a distinct cluster, whereas all other samples do not cluster into distinct populations, even at the preferred *K* = 3. Additionally, microsatellites generally perform well at fine spatial scales, but are limited in capturing all of the genomic diversity and therefore complex demographic scenarios, which may be the case for tigers at these broad spatial scales [65,73]. This is apparent in Kolipakam *et al*. [40], where their analysis at the pan-India scale shows only the NE as a distinct cluster at *K* = 3 and all other populations show mixed ancestry and do not resolve into distinct clusters at higher values of *K*, whereas their separate analysis of population structure within regions revealed sub-structuring into distinct clusters.

**Figure 4.**
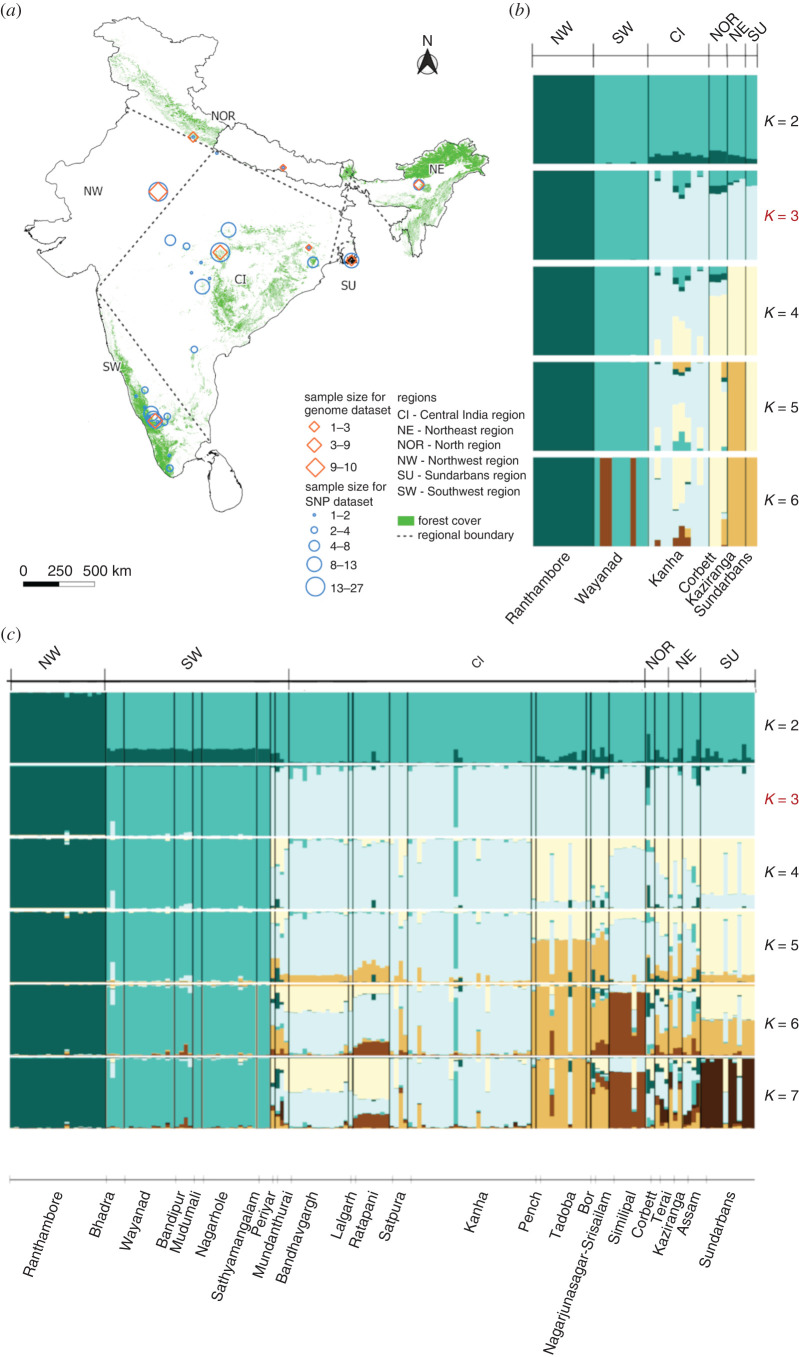
Population structure across India, which can be classified into six regions; northwest (NW), southwest (SW), Central India (CI), north (NOR), northeast (NE) and Sundarbans (SU); (*a*) shows sample locations of the two different datasets (table 1 row 1 and 3); whole-genome data from [33–36,38,39,44] and SNP data from [10]. (*b*) Population structure based on genome-wide SNPs from whole-genome sequence data. Results are shown for *K* = 2–6 for 37 individuals from eight protected areas that represent six regions (NW, *n* = 10, SW, *n* = 9, CI, *n* = 10, NOR, *n* = 3, NE, *n* = 3, SU, *n* = 2). (*c*) Population structure based on a panel of 81 SNP variants for 175 individuals from 24 protected areas (classified as tiger reserves), which represent six regions (NW, *n* = 21, SI, *n* = 52, CI, *n* = 78, NOR, *n* = 5, NE, *n* = 7, SU, *n* = 12). Results are shown for *K* = 2–7. Preferred *K* (based on the Evanno method [45]) is shown in red and is *K* = 3 for both structure plots. Labels above structure plots correspond to the geographic region, whereas labels below the structure plots are the protected areas where samples were collected. Specific protected area labels allow comparison of which of these overlap between the two datasets.

Challenges remain in resolving what the most probable population structure is for Bengal tigers within India. Typically, when resolving *K* to understand population structure, Δ*K* values should be considered in the context of what makes biological sense. Network analysis by Alcala *et al*. [65], using the dataset of approximately 10 000 SNPs from [12], suggested that the NW population separated first, followed by SW. This follows what we observe across SNP-based analyses, and the divergence of the NW is also revealed in Mondol *et al*. [41]. However, demographic history of Bengal tigers, modelled by Armstrong *et al*. [36], revealed earliest divergence of the NE population 3000–5000 years prior to divergence among all other populations. This divergence is potentially why we see greatest population differentiation between the NE and other populations in the mitogenome analysis (figure 4*b*). Tigers are known to exhibit female philopatry and male-biased dispersal therefore we may expect to see stronger structure in matrilineally inherited markers. This pattern of male-biased dispersal is common among mammals and results in patterns of nested mitochondrial DNA structure even when there is continued gene flow [74,75]. While this pattern of NE divergence reflects the inferences in Kolipakam *et al.* [40], PCA and structure analysis in Armstrong *et al.* [36] do not show this separation, and our genome-wide analysis indicates that the NE does not differentiate until higher *K*. This outcome and the shared ancestry between the NE and some CI populations in Kolipakam *et al.* [40] suggests that there has been continued gene flow between tigers in the NE and CI. Given that ‘Central India’ is large and extends almost as far east as Bangladesh, it is possible that tigers in these eastern states including Jharkhand, West Bengal and Odisha, could be connected by low levels of gene flow until recently to those in the NE. It is also possible that the lack of differentiation of the NE population in the SNP-based studies may be an artefact of uneven sampling across the populations; the NE is the least sampled region in these studies in terms of numbers of individuals and spatial distribution. However, when we sub-sampled all populations to *n* = 3 the Sundarbans and NE do not separate from other populations until *K* = 4 (electronic supplementary material, figure S3).

Inference of population structure based on Δ*K* only provides the most probable value of *K*, and there are often further levels of sub-structuring within regions that are important to detect in conservation of threatened species (e.g. [76]). Wild populations rarely fall into distinct units and the power required to detect structure is dependent on the scale of analysis and complexity of evolutionary history. Assessing structure at a broad, regional scale is useful to understand differences in recent demographic trajectories and evolutionary histories. However, conservation management is typically organized at a finer scale and can be dictated by funding, or geopolitical, or societal boundaries; therefore, detecting structure at finer scales where degree of structure is sometimes weaker is crucial in conservation genetics.

Fine-scale population structure and gene flow in Bengal tigers are best understood in the CI region, where multiple studies have attempted to resolve connectivity and population structure across this landscape [37,77,78]. Consistent patterns across a few microsatellite-based studies suggest that the region has multiple sub-populations with varying degrees of connectivity, supported by forest cover, but impeded by human disturbance and large highways [37,77,78]. Broadly, these studies indicate a central population cluster (Kanha, Pench, Melghat and Satpura), a Northern cluster (Bandhavgarh) and a Southern cluster (Tadoba). Despite the unclear clustering at the pan-India scale in Kolipakam *et al*. [40], they do show population structure within the CI landscape when assessing this as an individual unit. Also, this fine-scale pattern of structure emerges within CI, even when conducting a pan-India analysis using the SNP panel. Wider geographic sampling in [10] facilitated by non-invasive samples (figure 2*a*) revealed population structure within CI at higher *K* values (figure 2*c*). However, such sub-structuring remains absent from the genome-based analyses, as the spread of geographic sampling within CI is limited to a single population, Kanha tiger reserve (figure 2*a*). This re-iterates that continued study, by different groups, with varied samples and marker sets is ideal in conservation, because it allows unbiased replication, and inferences that are robust. Ideally, raw datasets should be made available so that samples and data over time can be combined, synthesized and re-analysed.

In our whole-genome analysis, *K* = 4 revealed separation of north-northeast-east India from populations in CI, while high values at *K* = 6 suggested population sub-structuring within SW India (figure 2*b*). In addition to population substructure within CI at *K* = 4, the SNP panel analysis also suggests some separation of Sundarbans and populations in the south of CI from the other populations. However, at this higher value of *K*, populations start to appear admixed rather than forming distinct clusters, which suggests shared ancestry among these populations and indicates very recent loss of connectivity within CI, which is likely due to anthropogenic landcover changes [37]. The easternmost population (Sundarbans) forms a separate cluster at *K* = 5 and Similipal Tiger Reserve (in eastern CI) separates at *K* = 6. Increasing *K* further shows population substructure within CI, with populations from North-Central (Bandhavgarh and Ratapani), Central (Satpura, Kanha and Pench), East (Sundarbans), East-Central (Similipal) and South-Central (Tadoba and Nagarjunasagar-Srisailam) all forming distinct clusters. However, the NE and the north become admixed at these higher values of *K* and increasing *K* may begin to reveal substructure that is not actually present within the sample regions.

Overall, the fine-scale differentiation between central-east–north-northeast India remains poorly understood. The north or the Terai habitats in the foothills of the Himalayas are one geographic region that remains relatively unresolved. Broader geographic sampling within the north and genome-wide genetic data to evaluate relative divergence between these populations and their demographic histories may be instructive in understanding the complex patterns of population structure here. Additionally, the population genetic structure of NE India tigers is inconsistent, and we are unable to resolve this unequivocally with the data we have so far. This may require additional sampling not only in NE India, but also internationally, in Myanmar and Bhutan to determine whether the NE harbours unique ancestral variation.

Going forward, studies of tigers must prioritize sampling across regions that remain under-represented. These regions include the NE, which likely harbours considerable genetic variation, the north across the Terai arc, and east including states such as Tamil Nadu, Andhra Pradesh, Telangana and Odisha. Increased sampling through the use of non-invasive approaches will aid in our understanding of connectivity between these eastern regions and CI, whereas in the NE, increased sample size with better spatial distribution can allow us to further investigate whether this region clusters with CI. Given the paucity of opportunities for collection of invasive samples from wild individuals, especially in areas with low tiger density, non-invasive sampling methods must be prioritized to improve representation from such regions. We have shown here that at the broad, regional scale reduced representation SNP panels can resolve population structure that is shown by many hundreds of thousands of SNPs.

### (c) Where we are in tiger conservation genetics

Here we have demonstrated how our understanding of population structure in Bengal tigers has changed depending on marker type and sampling strategy. While questions remain as to the most effective sampling strategy to understand connectivity among populations, we show that, when using SNP markers, fewer markers (in the thousands) can be as informative as whole-genome sequence data when assessing populations at a broad spatial scale, while much reduced numbers of SNPs can possibly resolve this structure but on occasion give spurious clustering. Denser spatial sampling within a region, even with few markers, can resolve structure at a local scale. This is especially useful given the challenges of sampling whole genomes in threatened species and for the importance of understanding local-scale connectivity for conservation management. Additionally, the availability of HTS data allows us to combine data across studies for synthesis and re-analysis, which is often difficult with older marker types.

At the onset of the genomics era *circa* 2000 when sequencing the human genome was accomplished, sampling whole genomes from threatened mammal species seemed almost intangible (aside from the great apes). Yet 20 years later, for tigers alone, there are now four different genome assemblies available, a scaffold-level assembly of the Amur subspecies [79], one chromosome-level assembly from the Malayan subspecies [36] and two different chromosome-level assemblies for the Bengal subspecies [80]. Not only have advances in molecular biology driven the scope of what is possible in conservation genomics, but this has expanded in parallel with computational biology. Pipelines for genome assembly and annotation mean that it is possible to generate reference genomes for non-model organisms with relative ease (e.g. supernova). Such resources allow characterization of genetic diversity and identification of variation among populations that are paramount to understanding and protecting biodiversity globally.

The utility of genomics to conservation that was predicted over 10 years ago [22] is starting to manifest; for example, the approach of sampling fewer individuals at far greater numbers of markers is shown in our analysis of whole-genome population structure, which is useful in understanding broad biogeographic histories. Aside from adding power to these somewhat rudimentary questions, the availability of whole-genome datasets has allowed researchers to address questions of inbreeding and selection. For example, Liu *et al*. [39] identified signatures of selection among tiger subspecies and revealed regions potentially under selection that may be responsible for smaller body size and darker coat colour in the Sumatran subspecies. Armstrong *et al*. [36] identified signatures of selection (potentially for cold adaptation) in Amur tigers. In the context of Indian tigers, there are populations that live in unique environments and are potentially under local selection. Tigers in the Sundarbans of the mangrove forests, for example, live in ecologically unique habitats thought to affect their diet and also have distinctive morphological characteristics [81]. In fact, Singh *et al*. [82] suggest these tigers should be an independent management unit. Our analyses (figure 2*b*,*c*) on whole genomes and 81 SNPs suggest that tigers here are genetically distinct. Additional analyses to investigate signatures of selection on candidate genes (those involved in salt metabolism, since the water here is brackish) or sliding window approaches that investigate selection will help better understand whether these tigers are indeed locally adapted and should be managed as a separate population.

### (d) Application of population genetics to conservation in the future

Assessment of population structure, especially at the local scale, will always be a central question in conservation. Practitioners use this information to make decisions on when and where efforts to protect or restore population connectivity should be placed, which is essential for conserving remaining diversity [24]. Studies that go beyond population structure to ascertain the impacts of the loss of connectivity and reduced population size on threatened species truly represent conservation genetics and will allow for prioritization of resources in conservation management. One application of genomics that has proved useful in conservation is a better understanding of inbreeding in wild populations. Small population sizes and reduced connectivity place these species at risk of inbreeding and accumulating deleterious alleles. Recent studies have highlighted the presence of long runs of homozygosity in individuals from specific populations, for instance in wolves [83] and mountain lions [84]. They also highlight strategies for mitigation, including identifying source populations for assisted translocation based on sharing of homozygous tracts. Khan *et al*. [38] quantified inbreeding and mutation load among the three (NW, south, central) tiger populations within India using whole genomes and showed that the individuals in the NW population, which separates first in most structure analyses, have higher inbreeding on average compared to other tigers in India. They also predict potential loss of function and deleterious alleles in the tiger genomes. Several recent studies highlight long runs of homozygosity (ROH) and high mutational load in endangered species (e.g. kakapo [85]; rattle snakes [86]; wolves [83]; killer whales [87]), while some studies show that decreased survival and population growth rate are correlated with higher inbreeding and mutation load (e.g. see arctic foxes [88] and alpine ibex [89]). Unfortunately, our understanding of the functional effects of specific mutations and their impacts on fitness in endangered species remains poor.

While advances in our understanding and application of population genetics over the past 50 years have opened up the possibility for conservation genomics, these advantages are not being realized across many endangered taxa. Tigers have been at the forefront of conservation efforts since the 1970s when population genetics was just starting out as an empirical field [90]. We have seen genetic research on tigers develop as it became possible to apply these methods and approaches to address questions in wild mammal species. While it has been possible to develop genomic-scale resources for this species, numerous, less charismatic species that are not under the conservation spotlight do not get the same attention or funding [91]. While the use of flagship species is in many ways beneficial to conservation, studies on the diversity of fauna within these ecosystems are required to understand the threats to different species and to monitor the effectiveness of any conservation measures that are put in place. Therefore, the use of the approaches for non-invasive sampling and reduced yet informative marker sets will likely be of huge importance in effective conservation and management of biodiversity globally. Independent, repeated assessments of population structure within tigers are helping to build a cohesive picture of tiger population genetics that is moving our understanding of this species forward. As with all scientific endeavour, repeated analyses will convince us which populations/landscapes need more study/prioritization for conservation. The challenge will be to ensure that these studies provide adequate information content to not simply describe population genetics, but to enable conservation prioritization of small and isolated populations.

## Data Availability

All data included in this manuscript were generated by previous publications and are available on NCBI National Centre for Biotechology Information (see https://www.ncbi.nlm.nih.gov/).
